# Informational continuity by skilled birth attendants during antenatal care in Lesotho

**DOI:** 10.4102/hsag.v29i0.2403

**Published:** 2024-01-22

**Authors:** Angelina Zhangazha, Doreen K.M. Kaura, Anneline E. Robertson

**Affiliations:** 1 Department of Nursing and Midwifery, Faculty of Medicine and Health Science, Stellenbosch University, Cape Town, South Africa

**Keywords:** informational continuity, continuity of care, antenatal care, primary healthcare, coordination of care

## Abstract

**Background:**

Informational continuity (IC) is one of the four approaches that enables integrated people-centered health services. IC enables the availability of all health and psychosocial information of the pregnant women at all health encounters. World Health Organization (WHO) recognised that ineffective IC results in fragmented health care and duplication of services. Hence, IC may assist in the reduction of maternal morbidity and mortality.

**Aim:**

The purpose of this study was to explore and describe the experiences of skilled birth attendants (SBAs) with IC during the antenatal period

**Setting:**

Three primary healthcare centers in Maseru district, Lesotho.

**Methods:**

A qualitative descriptive phenomenological design was used with purposive sampling to choose nine participants.

**Results:**

Four themes emerged; Theme one: SBAs and pregnant women information communication, theme two: Information communication between the SBAs, theme three: information collection during ANC and theme four: guidelines used during ANC to standardise care. Several challenges regarding information communication form the sources of information, transition of information, information between caregivers and women which demonstrated the frustration between the women and the SBAs during ANC leading to ineffective care coordination.

**Conclusion:**

Enabling IC during ANC enables effective data collection from the sources of information, transition of information during care giving within and between health facilities.

**Contribution:**

Effective informational continuity enables effective care coordination in ANC in Lesotho.

## Introduction

The World Health Organization (WHO) has recognised Informational Continuity (IC) as one of the measures that may assist in the reduction of maternal mortality by increasing coordination of care, reducing duplication of services and fragmentation of care for pregnant women (WHO 2018:9). Lesotho did not meet the 2015 millennium development goals and was still struggling to meet the sustainable development goals and the decrease in maternal mortality was very low (Lesotho Maternal Deaths report 2021:6). Furthermore, on review of the maternal mortality, in Lesotho, it was noted that lack of information and delays in seeking help were among some of the avoidable factors during pregnancy.

Informational continuity in pregnancy is the sharing of information on prior events and current circumstances to ensure that care provided is appropriate for the woman and her current condition (Andres et al. 2016:116). This approach ensures that information about pregnant women is readily available to healthcare providers for continuous care (Sandall 2014:8). Hudson et al. (2019:342) asserted that the sharing of information between individuals, families and healthcare providers enhances the coordination of care for all patients. Transition of information is important as the pregnant women are often not seen by the same healthcare provider at a facility and they also move from one area to another. Inadequate and/or inaccurate patient information causes fragmentation in care, as asserted by M’Rithaa, Korpela and De la Harpe (2015) and Mostert-Phipps, Pottas and Korpela (2012:330). Haggert, Roberge, Freeman and Beaulieu (2013:1220) and Sellers (2017:17) indicated that IC allows pregnant women to participate in their care and this will facilitate early identification of problems. Murray et al. (2019:9) asserted that care for pregnant women is improved by a multidisciplinary approach, as SBAs collaborate with other healthcare providers.

A qualitative study was conducted in the Volta region of Ghana, which aimed to measure continuity of care and fragmentation of care during ANC. The study revealed that fragmentation of care was very high as women often changed between facilities during the perinatal period (Dery 2017:158). A qualitative study in Australia conducted by Gardner et al. (2013:34) mentioned that if pregnant women carry their case notes with them as they change facilities, it will enable an improved transition of information. The authors further revealed that shared information within primary healthcare reduced duplication of care and provided health professionals with the opportunity to access records of care from other healthcare providers.

The WHO framework for Integrated People-centred Health services (IPCH) was used as the conceptual framework for this study (WHO 2018:20). Furthermore, WHO states that IC is the primary facilitator of care coordination. In this framework, there is parallel coordination involving communication within the PHC centres, sequential coordination involving communication with the next level of care and system enablers that involves the use of records, policies and guidelines as a form of communication (WHO 2018:20).

**FIGURE 1:**
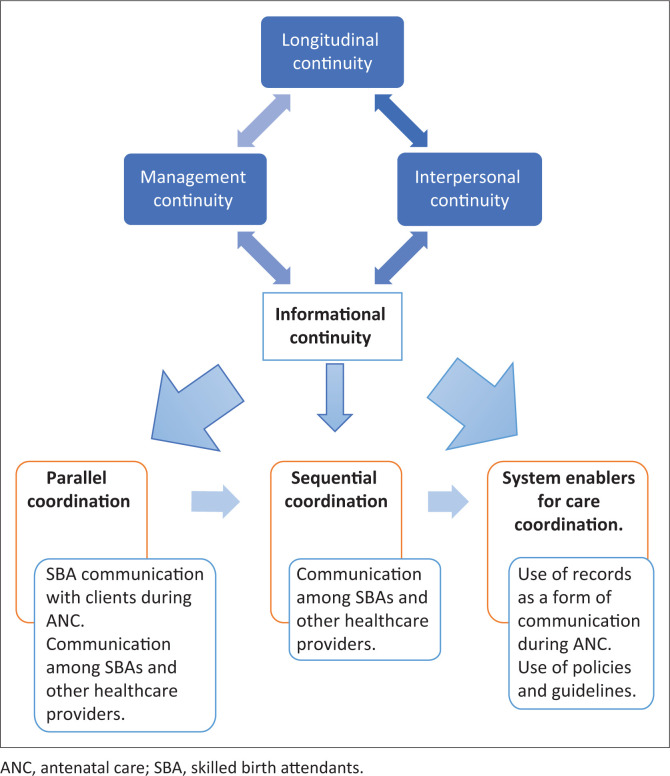
World Health Organization framework (WHO 2018) with research objectives.

Informational continuity is one of the various approaches to continuity of care developed by the WHO (WHO 2018:17). World Health Organization (2018:17) further described interpersonal continuity as the subjective experience of the caring relationship between a patient and his or her health care professional. Longitudinal continuity is described as a history of interaction with the same healthcare professional in a series of discrete episodes, whereas management continuity is described as the effective collaboration of teams across care boundaries to provide seamless care (WHO 2018:17). Informational continuity on the other hand is described as the availability of clinical and psychosocial information at all encounters with professionals (WHO 2018:17). Figure 1 shows the WHO framework on IPCH services approaches and interventions for achieving continuity of care and care coordination (WHO 2018:8).

There are no published research reports on IC during ANC in Lesotho. The purpose of the study was to explore and describe the experiences of SBAs with IC within the PHC settings in Maseru, Lesotho. The objectives of the study were to: (1) explore and describe the experiences of the SBAs on communication with pregnant women, (2) explore and describe the experiences of SBAs on communication among themselves and other healthcare providers, (3) to explore and describe the experiences of SBAs with the use of records as a form of communication and (4) to explore and describe the experiences of SBAs with protocols and guidelines during ANC.

## Problem statement

World Health Organization factsheet (2023) indicated that maternal mortality remained high with about 287 000 women dying during pregnancy and after childbirth in 2020. Additionally, WHO states that 95% of all these maternal deaths occurred in low- and middle-income countries with most of the maternal deaths caused by preventable factors. Informational continuity could be one of the measures that could prevent maternal mortality, as poor transition of information and fragmented care result in poor IC (WHO 2018:9). The researcher assumed that if there was IC among SBAs, there would be effective continuity and coordination of care.

## Research methods and design

### Designs

A qualitative descriptive and phenomenological approach was used for this research study. The approach allowed the SBAs to share their experiences with IC during the ANC period. Individual semi-structured interviews were conducted to collect data. This research study was based on Husserl’s philosophy, which persisted in describing the experiences of the SBAs, based on their worldview and on setting aside all personal beliefs using bracketing (Polit & Beck 2017:471). Furthermore, data analysis was done using Colaizzi’s seven-step framework (Morrow, Rodriguez & King 2015:643).

### Context

The study setting for this research was three primary healthcare centres (PHCs) within the district of Maseru, Lesotho. Lesotho is a small, mountainous lower middle-income country of 11 720 square miles, enclaved by the Republic of South Africa, with a population of around two million people (Lesotho Demographic Health Survey [LHDS] 2014:7). Lesotho consists of ten districts, namely Maseru, Mafeteng, Quthing, Qacha’s Nek, Thaba Tseka, Mokhotlong, Berea, Butha Buthe, Mohale’s Hoek and Leribe (LDHS 2014:7). Furthermore, Lesotho has a total of 286 health facilities, of which 265 are PHC facilities, 20 are hospitals providing secondary health care and one tertiary hospital. The Christian Health Association of Lesotho (CHAL) is a non-governmental organisation, which manages 71 PHC centres, eight district hospitals and four teaching hospitals. The other facilities are managed by the Government of Lesotho (GoL). Christian Health Association of Lesotho is supported by the GoL through the Ministry of Health though it is managed independently (Master Health Facility List 2020). The PHC centres in Lesotho provide ANC for pregnant women and SBAs assist in uncomplicated childbirths in these PHC facilities.

This study was conducted in the Maseru district, Lesotho. Maseru is the capital city of Lesotho and is divided into Maseru urban and Maseru rural. Maseru urban has a tertiary hospital with four filter clinics that allow referral of pregnant women directly to the tertiary hospital. There are 13 PHC centres and one tertiary hospital in Maseru urban. Maseru rural has two secondary health facilities, which are the referral centres for the 20 PHCs (Master Health Facility list 2020). This study was conducted in two urban PHC facilities where there is a high-density attendance of pregnant women and one rural PHC setting where there is a low-density attendance of pregnant women. The rural PHC centres had a monthly average of 20 new ANC bookings and 80 subsequent ANC contacts. The first urban PHC had a monthly average of 40 new ANC bookings and 280 subsequent contacts, whereas the second urban PHC had an average of 90 new ANC bookings and 300 subsequent contacts.

### Study population and sampling strategy

The Maseru urban PHC centres have a total of 18 SBAs, five work permanently in the Mother and Child Health (MCH) clinics, which include ANC and labour unit, and 13 SBAs work within the outpatients’ department in the PHC centre. The rural PHC centre has three SBAs that rotate between the MCH and outpatient clinics within the PHC centre.

Participants were selected for the study utilising a purposive sampling method to select those who had worked at the PHCs for 2 years or more as they had sufficient knowledge of informational processes. Skilled birth attendants who were not working at the antenatal clinic were excluded from the study.

### Data collection

Data collection was done from March 2021 to May 2021. A semi-structured interview guide was drawn up in English, which is the official language used by SBAs in Lesotho. The semi-structured interview guide was drawn up by the first researcher with the guidance of the second researcher, based on the research objectives as shown in Table 1.

**TABLE 1:** Semi-structured interview guide.

Research objective	Questions asked
To explore the experiences of SBAs on communication with pregnant women during ANC.	Tell me about your experience with communication with pregnant women
To explore the experiences of SBAs on communication among themselves and with other healthcare providers.	Let’s talk about your experience with information sharing within teams
To explore the experiences of SBAs with the use of records as a form of communication during the ANC	Let’s talk about your experience with sharing records during ANC
To explore the experiences of SBAs with protocols and guidelines during ANC.	Tell me your experiences with protocols and guidelines

SBA, skilled birth attendants; ANC, antenatal care.

Data saturation was achieved at the sixth interview; however, the first researcher continued with interviews until the ninth participant. Nine participants were interviewed to ensure that an equal distribution of participants was selected from both the rural and urban PHC centres. The first researcher is a midwife who is employed as an educator and therefore had no prior contact with the participants.

The first researcher visited the various PHC facilities to introduce herself and provide information about the study. Permission to enter the PHC centre was sought from the security guard and to conduct the interviews with the nurse in charge of the PHC centres. A participant information leaflet was given to each participant before they provided written consent. Copies of ethical clearance letters were given to the nurse in charge of the PHC centres for record keeping. Two pilot interviews were conducted as per inclusion criteria to refine the interview guide and interview skills of the first researcher. Interviews were scheduled according to the availability of the participants on the date, time and venue convenient for the participants. Following the signing of the informed consent, the interviews were conducted in a private room. Three participants from each of the urban PHC facilities, and three in the rural PHC centres, met the inclusion criteria and all agreed to participate in the interview.

The first researcher requested the participants to switch off their cell phones during the interview to avoid further distractions. Permission for recording of the interview was obtained from each participant, and the interview process took between 40 min and 60 min per participant.

### Data analysis

The interviews were transcribed and analysed using Colaizzi’s seven-step (1978) framework (Morrow et al. 2015:643). The audio recordings were transcribed by the first researcher. The interviews and the transcripts were read and listened to by the researchers to familiarise themselves with the data. Significant statements were extracted from each transcript. Meanings were formulated as they emerged. The formulated meanings were organised into clusters of themes and categories. The clusters of themes were validated by referring to the original transcript to ensure that no data had been ignored. The results were integrated into an exhaustive description and essential structure of the phenomenon being studied.

### Ethical considerations

Ethical clearance was obtained from Stelenbosch University (HREC) and Lesotho, Ministry of Health National Research and Ethics Committee. S20/10/270 and Lesotho-ID128-2020. Further permission to conduct interviews at the PHCs was given by the Maseru District Health Management Team. Ethical considerations observed during data collection were the right to self-determination, the right to confidentiality and anonymity and the right to protection from harm. Participants were not reimbursed, but they were given a token of appreciation in the form of refreshments of R10 000 per participant for their time.

### Trustworthiness

The criteria used to ensure trustworthiness were credibility, transferability, dependability and confirmability according to Guba and Lincoln 1985 as cited by Cypress (2017:256). Purposive sampling ensured participants could provide detailed information, thus allowing the participants’ own words to be cited. Bracketing was applied, peer debriefing was done through discussions between researchers, and co-coding was performed. Data collection was pursued until data saturation was achieved to provide a detailed rich description of the phenomenon (Cypress 2017:256–258). Member checking was performed by taking the findings back to the participants for verification and the participants agreed with them and no new information was added.

## Results

The data analysis yielded several formulated meanings and sub-themes. Four key findings emerged, namely theme one, sharing information during the antenatal period; theme two, information transition during the ANC; theme three, challenges to information continuity during the antenatal period and theme four, challenges with the use of protocols and guidelines.

### Demographic characteristics of participants

A total of nine participants were interviewed. The participants comprised two males and seven females ranging between ages 30 and 53 years old. Eight of the participants were married with children and one was single with no children. The total number of years of service ranged from 5 to 22 years. The participants worked in MCH for a period ranging from 3 to 22 years. Two of the participants were Nurse Clinicians, one was a Nursing Officer and six were Registered Nurse midwives.

#### Theme one: Sharing information during the antenatal period

The categories from theme one included the involvement of the partner in information sharing, the information provided by the woman and information obtained from the examination.

**Category 1.1: Involvement of the partner in information sharing:** Family support and companionship were expressed as vital to enable women to confidently know about their care and involve their partners when the care is changing. During pregnancy, women require support because of the physiological and psychological changes of pregnancy. The partner and family of a pregnant woman play an essential role in supporting a pregnant woman, particularly when a problem arises. The participant mentioned:

‘If there are problems, we have to include the third person being the family member or the village health worker to support the pregnant women.’ (Participant 1, 34 years, female, registered nurse midwife)

Pregnant women are encouraged to come with their partners to ANC, even though most of the community objects to this and some of the pregnant women have husbands who work out of the country. One participant mentioned:

‘… and we also advise them to come with their partners when they come for ANC so that while we are giving this woman health education the partner will be listening so that they know how to take care of this pregnant woman at home.’ (Participant 9, 37 years, female, registered nurse midwife)

**Category 1.2: Information sharing with pregnant women:** Informational continuity during pregnancy can be obtained through the sharing of information during ANC. Participants indicated that the information sharing starts during the pre-pregnancy period when the pregnant women are equipped with knowledge so that when they fall pregnant, they will report for ANC early in the pregnancy. One participant mentioned:

‘Basically, I would say the antenatal period is a period which does not only start when a woman comes in pregnant but is continuous care from when the patient is attending family planning. We start preparing them for future pregnancies during this time.’ (Participant 2, 54 years, male, registered nurse midwife)

Antenatal care also included providing health education about maintaining a healthy pregnancy and labour including the danger signs of pregnancy.

**Category 1.3: Information obtained from physical examination:** Information sharing during pregnancy occurs as the SBAs provide ANC to women. The ANC included obtaining the medical, surgical family and psychosocial history, and performing examinations to identify risk factors. One participant mentioned:

‘… So, after that we examine them, we give them health education about the complications of pregnancy, signs of labour, also the danger signs in pregnancy so that when they see these danger signs, they should come to the health centre.’ (Participant 7, 30 years, male, registered nurse midwife)

**Category 1.4: Information provided by the woman:** The SBAs asserted that following the examination of the woman, they would get the history of the woman privately especially based on sensitive issues such as intimate partner violence. Two participants mentioned:

‘Eee apart from that we also do our examination where we take that woman to the examination room. Then we might also get some history that she was hiding, for example, if the woman was abused, we also observe if there are any signs of that.’ (Participant 3, 46 years, female, nurse clinician)

‘… some of the pregnant women do not give accurate information even if they have been asked.’ (Participant 4, 39 years, female, nurse clinician)

During this therapeutic communication, the SBAs would also observe for signs of abuse of the pregnant women. This was in addition to the information received from the pregnant woman, the physical examination and investigations conducted.

#### Theme two: Transition of information during antenatal care to enable continuity

Categories that emerged from this theme were transition of information within the facility, transitioning of information within the community enabled, transitioning of information communication between facilities, information transition necessary between preconception and ANC and communication documents during ANC.

**Category 2.1: Transition of information within the facility:** The exchange of information within the PHC occurs using records, meetings and reports. The SBAs communicated with each other to make decisions about the management of pregnant women. Participants mentioned:

‘So, the communication between midwives, maybe let’s say I am consulting the ANC, maybe one of the patients has high blood pressure I will call my colleague so that we discuss the patient situation and come up with a possible solution.’ (Participant 6, 32 years, female, registered nurse midwife)

Furthermore, sharing of information within the facilities is done during the weekly meetings. However, in case of urgent matters that cannot wait for weekly meetings, the SBAs call urgent meetings and discuss the matters as necessary. One participant mentioned:

‘Eee sharing of information here at MCH, we normally have weekly meetings here or sometimes we cannot even wait for the weekly meeting if maybe there is something alarming, we can meet at that time as midwives to discuss the matter.’ (Participant 5, 37 years, female, registered nurse midwife)

**Category 2.2: Transitioning of information with the community:** Transitioning of information with the community enabled successful care of pregnant women. An informed community was the source of support for the family and community level. Participants indicated that information in the community was achieved mostly through health education sessions. One of the participants said:

‘Much as we share information by doing pitso’s (health education) in the community talking to pregnant women and women in general we should never assume that they know it all.’ (Participant 2, 54 years, male, registered nurse midwife)

**Category 2.3: Transitioning of information communication between facilities:** The PHC centres manage pregnant women who present with no complications. In the event of a complication, the pregnant woman will be referred to the district or tertiary hospital. Feedback is important for the effectiveness of IC as it allows the SBAs to know if the pregnant women was managed appropriately. There was poor feedback from the referral hospitals on how the pregnant women were managed as well as their outcomes. Participants mentioned:

‘About the pregnant women we refer to the hospital, sometimes we never get feedback on how they progressed but most of the time we meet them during six weeks’ postnatal clinic. Sometimes we see them when they come for OPD, for instance, there is one we referred to the hospital, she miscarried but she didn’t come immediately after she was discharged, I only met her when she came for OPD here and I asked, what happened. She said ahh ‘Me I met problems there, I didn’t make it. That is how we get the feedback and how we meet them.’ (Participant 3, 46 years, female, nurse clinician)

‘Otherwise, the hospital does not call to give us feedback of the outcomes of the patient. So somehow it is a challenge because we also need to be appreciated when we have made a good decision.’ (Participant 7, 30 years, male, registered nurse midwife)

**Category 2.4: Information transition necessary between preconception and antenatal care:** Information evolved during ANC as a necessary transition from preconception to pregnancy, from SBA to pregnant women, between SBAs, during meetings, through health education, during referrals and documentation using Lesotho obstetric record (LOR) and institutional records such as ANC register, E-register, tally sheet and manual appointment register. Participants mentioned:

‘We have a Lesotho obstetric record which I think it contains all the information that is necessary to everyone that is going to assess the client. If that record is always there, I think everybody who is going to meet the client is going to have information. So even if I am not present at the moment and the client has been seen, the next time I see the patient with LOR I am going to get the information in the record.’ (Participant 1, 34 years, female, registered nurse midwife)

‘So, ee during the clinic we have three kinds of registers. They are different because there is the LOR and manual ANC register together with the E-registers. In these registers, we record the same information inclusive of blood results.’ (Participant 4, 39 years, female, nurse clinician)

**Category 2.5: Communications documents during antenatal care:** There are different types of documents used during ANC. These documents include Bukana, LOR, the Antenatal register, the appointment register, the E-register and tally sheets. Bukana is a small booklet that is used by all adults for outpatient department consultations. During ANC, the Bukana is used to record investigations. One participant mentioned:

‘We also use the bukana to record TB (tuberculosis) and Covid 19 screening as well as all the antenatal investigations from urinalysis and the blood tests.’ (Participant 6, 32 years, female, registered nurse midwife)

The LOR is used to record all the care given to the pregnant woman during pregnancy inclusive of findings during examinations, which is accessible to everyone going to see the clients. One participant mentioned:

‘We have a Lesotho obstetric record which I think it contains all the information that is necessary to everyone that is going to assess the client. If that record is always there, I think everybody who is going to meet the client is going to have information.’ (Participant 1, 34 years, female, registered nurse midwife)

The summary of all findings of the history taking, examination, investigations, health education and treatment is written in the ANC register, which is updated on every ANC visit. A daily tally sheet is used by all the PHC centres as a statistical tool to monitor the daily attendance of pregnant women. One participant mentioned:

‘Ee we also apart from the LOR we have ANC register. So, on the register, we write those women and also, they are updated at every visit. Or if she has some results that may come, and she is not there. So, we just write the results in the Register so that when we update her during subsequent visits we also update in the LOR.’ (Participant 7, 30 years, male, registered nurse midwife)

The E-register, which is an electronic register, was used to capture information from the LOR and the ANC register. The E-register also has an appointment system used to book subsequent ANC appointments. One urban and one rural PHC facility had the E- register. One of the urban PHC centres did not have an E-register. Two participants mentioned:

‘Apart from that we use the E-register this one is also used to help us to see if the women have missed the appointment. So, we also have the same information about that woman. It also contains the same information from the LOR.’ (Participant 7, 30 years, male, registered nurse midwife)

‘We also use the E-register to book subsequent appointments for pregnant mothers. We can trace them if they don’t come.’ (Participant 1, 34 years, female, registered nurse midwife)

The manual appointment book is also used to help the SBAs to trace the missed appointments. The manual appointment book further acts as a tally sheet, which indicates the type of care the women have received. The participants mentioned:

‘We also book our clients in the appointment book so that when they don’t come, we send the village health workers to trace them.’ (Participant 9, 37 years, female, registered nurse midwife)

‘… then we also have the tally sheet. The tally sheet will also include if they have received supplements or not, whether she was tested for syphilis, and again we have the PMTCT register.’ (Participant 5, 37 years, female, registered nurse midwife)

There are in essence a lot of documents used in the institutions for documentation, which could be integrated through a single electronic record.

#### Theme three: Challenges to information continuity during the antenatal period

It emerged that the SBAs felt that there was fragmentation of care within and between the health facilities, which included the interruption in the care of women, prolonged waiting time affecting the flow of information and inadequate and or incorrect information given by pregnant women. Furthermore, the lack of protocols for the transition of information, interruption in the care of women and prolonged waiting time affected the flow of information.

Categories that emerged with this theme were challenges with communication and challenges with documentation.

**Category 3.1: Challenges with communication:** Interruption of the care of women caused communication challenges as well as frustration for the SBAs. If the communication challenges are not overcome, the pregnant woman might not experience a positive pregnancy outcome. Some of these challenges are caused by multitasking by the SBAs, prolonged waiting time and inadequate or inaccurate information received from pregnant women. One participant mentioned:

‘At times when you are attending the ANC clients and a labouring woman arrives, you must leave the pregnant woman and attend to her. This prolongs waiting time for the women.’ (Participant 2, 54 years, male, registered nurse midwife)

Pregnant women experienced prolonged waiting times during the antenatal booking, which later resulted in delayed antenatal booking with subsequent pregnancies. One participant mentioned:

‘I think these women delay coming for the initial visit because of the time that they take here during the initial booking.’ (Participant 2, 54years, male, registered nurse midwife)

The lack of trust between the SBAs and the pregnant women was expressed as the reason for the women to withhold information such as the human immunodeficiency virus (HIV) status. Some of the women might have defaulted the HIV treatment. Furthermore, they would not even give enough information regarding the number of pregnancies, which is necessary for further management. One participant mentioned:

‘Some of these women do not give us all the information that we need, more especially their information related to their HIV status. They may have tested and started treatment and defaulted and when they come here, they come as new patients. Some of them don’t give us enough information about the number of pregnancies they have had.’ (Participant 8, 42 years, female, nursing officer)

Challenges with the collection of information and documentation of the given information often create hurdles in the flow of communication between pregnant women, SBAs and health facilities.

**Category 3.2: Challenges with documentation:** Incomplete documentation, coupled with inaccurate information, was expressed as the main cause of fragmentation of care. Incomplete documentation may lead to inaccurate information because of SBAs being too lazy to complete information in the documents. One of the participants mentioned:

‘The one challenge that we have as midwives is that some of us will be lazy to write you find them writing nothing abnormal detected under physical examination so that does not give us the information that we need.’ (Participant 8, 42 years, female, nursing officer)

The SBAs stated that they would use one document, which is the LOR to collect information from the women. Tests and investigations were recorded in the LOR as well as in the Bukana. Demographic information would further be recorded in the ANC register. One participant mentioned:

‘So first of all, while we are taking the history we document in the Lesotho obstetric record, then the tests are also recorded in the Bukana and then from there we have the ANC register where we record the names, the gestational age, the Hb of this woman, the HIV status, whether she is on ART or not, all that information.’ (Participant 5, 37 years, female, registered nurse midwife)

This duplication of information and documents caused fragmentation of care and frustration among SBAs as it increased the loss of flow of information.

#### Theme four: Challenges with the use of protocols and guidelines

**Category 4.1: Guidelines as used for communication:**Guidelines are recommendations that direct care within the health system, whereas protocols provide information on the best care and management of pregnant women. Protocols for the management of most pregnancy complications are included in the Lesotho antenatal guidelines. Protocols were present at the healthcare centres as charts with protocol management. One of the participants mentioned:

‘No, we don’t have a specific protocol here at the health centre but usually when we have any abnormality, we do have phone numbers for the doctors that we refer to.’ (Participant 1, 34 years, female, registered nurse midwife)

The participants were not sure if they had protocols at their health centres, as they had indicated that there were no clear protocols at the health centres. The SBAs were aware of the ANC guidelines though it was difficult to follow them because of the late booking by pregnant women. One of the participants mentioned:

‘We have ANC guidelines. The guidelines tell us when to see pregnant women. For example, we see them as soon as they miss their period and then they come back at 24–26 weeks then they come for 32 weeks and 36 weeks.’ (Participant 6, 32 years, female, registered nurse midwife)

The protocols and guidelines are disseminated through workshops. These workshops are often not attended by all the SBAs but rather by one person who would then give feedback on important information to others. One participant mentioned:

‘Usually when one goes to a workshop you come and do a step-down training to the others who did not go.’ (Participant 8, 42 years, female, nursing officer)

Challenges with IC during the antenatal period were caused by poor documentation, miscommunication of information, and more importantly inadequate and or lack of dissemination of standardised protocols and guidelines.

## Discussion of findings

**FIGURE 2:**
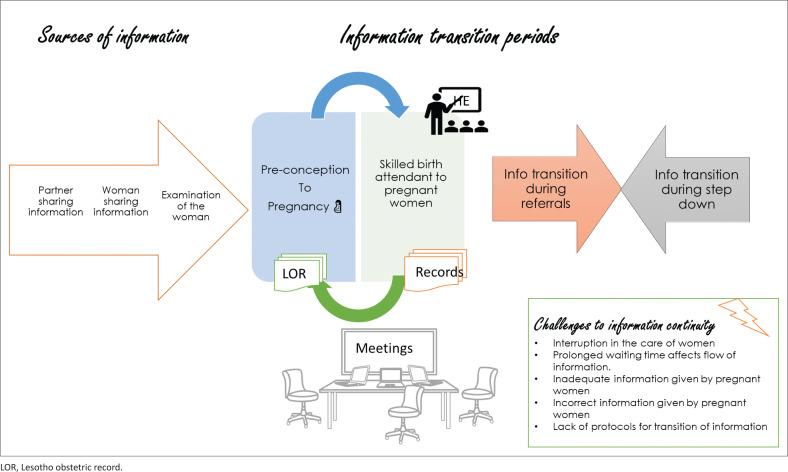
Summary of findings.

Figure 2 summarises the findings of the study. The participants expressed that IC during the antenatal period was enabled by the sources of information, which included the woman, her partner or companion, examination of the woman and investigations during antenatal visits. The transition of information occurred during the meetings, from the women’s obstetric records and the records kept at the institutions (ANC register, E-register, tally sheet and manual appointment register). Further, the transitions of information occurred during the referral of women to higher levels of care and the step down of information from higher levels of care to lower levels of care.

Several challenges were identified that influenced the IC, which included interruption in the care of women, prolonged waiting time that affects the flow of information, inadequate and or incorrect information given by pregnant women and the lack of protocols for the transition of information within and between health care facilities.

The key findings of this study were sharing of information, and information transition during ANC emerged as important aspects that enable IC for effective care coordination in Maseru Lesotho.

However, challenges with information communication would hinder effective coordination of care. The study highlighted that communicating with pregnant women through interaction during the first and subsequent ANC visits was the necessary point of sharing information with women. The interaction included obtaining a comprehensive history of the pregnant woman, examination and providing health education. Lesotho ANC guidelines (2020:15) and WHO (2016:112) state that a general physical examination and abdominal assessment should be done at first and subsequent contacts. The WHO (2016:105) and the Lesotho ANC guidelines (2020:10) stated that a comprehensive history should be collected during the first contact with pregnant women. Information was also communicated to pregnant women through health education. Health education was found to be very important during the first antenatal visits (Al-Ateeq & Al-Rusaiess 2015:240) and the WHO (2016:106) stated that improved communication during ANC visits led to a positive pregnancy outcome.

Family and or partner support with information sharing emerged as an important component in the care of pregnant women. Wong-Cornall et al. (2017:4) mentioned that family members appreciated the information they got from health providers, as it assisted with the continuity of care of the pregnant women at home or for further care. Partner involvement was also encouraged. However, most women were not accompanied during ANC as most partners were working out of the country. Kabanga et al. (2019:5) and Vermeulen et al. (2016:7) claimed that despite encouraging partner involvement during the ANC period, most males did not accompany their women to ANC.

The study revealed that multitasking of the SBA led to a prolonged waiting time for pregnant women. A study conducted in South Africa indicated that prolonged time taken during physical examination during a first contact could increase waiting times. If one of the SBAs attends to a pregnant woman on referral (Baron & Kaura 2021), the remaining one will be overwhelmed with work leading to prolonged waiting time for pregnant women, which the participants thought could lead to delayed booking with subsequent pregnancies. In both follow-up and booking appointments, the duplicate documentation increases the burden of work and extends consultation times. This leads to prolonged waiting time, which in turn leads to late antenatal bookings in subsequent pregnancies. Some of the pregnant women did not provide adequate information at the initial booking.

Transition of information was an important component between healthcare providers, and this improved the coordination of care for pregnant women. The SBAs communicated with each other while providing ANC, through meetings as well as through the LOR and the ANC register. The WHO (2018:20) encouraged teamwork among healthcare providers as this would promote collective teamwork. Further information transitioned within the community through health education. The WHO (2015:4) encourages community involvement by mobilising women through information provision, as this would improve maternal and newborn health through the support given to pregnant women.

Additionally, information transitioned to higher levels of care through referral systems following complications that needed further management. This was described in a study by Van Stenus et al. (2020:7) as handing off care and responsibilities between care providers. However, there was poor transitioning of information from the higher levels to the lower levels. This lack of feedback was perceived by seven of the SBAs as a lack of collaboration and fragmentation of information. Mostert-Phipps et al. (2012:327) asserted that discharge letters were not sent at all or not received in time at the primary healthcare level to provide informed healthcare. This supports the lack of communication between higher and lower levels of care.

Transitioning of information through documentation in this study revealed that there were several documents used during ANC. The documents included LOR, Bukana, ANC register, E-register, appointment register and tally sheets. Kuursisto, Asikainen and Saranto (2019:670) revealed the types of documents used during ANC as registers, obstetric records and E-registers which when utilised effectively would facilitate improvement in the care of pregnant women.

However, in this study the participants stated that multiple documentation was the main culprit in the errors in documentation, as well as increasing their workload. Similar information was documented in the LOR, the antenatal register and the E-register. An article by Mostert-Phipps et al. (2012:236) on improving continuity of care using electronic records in a South African perspective mentioned that the utilisation of only paper-based records was not adequate on its own; multiple documentation provided backup, even though it caused duplication of work.

This study revealed that representatives from the PHC centres would attend a workshop on the dissemination of guidelines and then give a step down to the rest of the SBAs. Attendance by one person was not enough as indicated by the lack of knowledge of what was contained in the guidelines. In South Africa, the health ministry was responsible for disseminating information on the updated ANC package to over 3000 public healthcare facilities throughout the country. This was revealed in a study on Implementing ANC recommendations by Hlongwane et al. (2021:221). All the SBAs must be trained on new guidelines as stated in the WHO’s framework for IPCHS states that collaborative training and education of the staff at the PHC is needed for them to improve skills and provide competence to fulfil their roles (WHO 2018:19–20).

### Strengths

The descriptive phenomenology allowed the researchers to perform in-depth interviews to describe their experiences. Analysis of the data using Colaizzi’s framework assisted in providing an in-depth description of the experiences of the SBAs. This framework has a step for validation of results with the participants and this was ensured therefore making the results more valid.

### Implications and recommendations

The SBAs in the study mentioned that when referring pregnant women to the next level of care, they do not often receive feedback from the referral hospital. The researchers recommend a referral policy that stipulates the feedback system within the healthcare centres as this assists with improvement in the management of antenatal women. Gaps in IC can be minimised using the referral policy, which was not available. The researchers also identified a need for electronic records in all PHC facilities as this may assist in the retrieval of information, communication and better coordination among the facilities.

## Conclusion

The findings of the study revealed that there was a two-way communication between the SBAs and the pregnant women. Informational continuity was achieved through history taking, examination, investigations and health education. However, most women were not accompanied by their partners to ANC. Multitasking and/or work overload by the SBAs led to prolonged waiting times for pregnant women. Some of the pregnant women did not give accurate and/or complete information, which would lead to mismanaging of the women during ANC.

Informational continuity was achieved through communication within the PHC centres during meetings. Communication with the community was mostly through health education. Communication with the referral hospitals was mostly through the referral written in the LOR or Bukana. It was revealed that there is poor feedback from the referral hospitals on the management given to the referred pregnant women was noted as a gap in IC.

Challenges with sharing information, transitioning of information within and between facilities, dissemination and use of standardised policies and guidelines and documentation of information require urgent solutions to enable continuity for effective care coordination during ANC.
